# On the estimation of total muscle work done in human walking

**DOI:** 10.1242/jeb.250352

**Published:** 2025-06-13

**Authors:** Gertjan Ettema, Jørgen Danielsen, Vemund Øvstehage, Knut Skovereng

**Affiliations:** Centre for Elite Sports Research, Department of Neuromedicine and Movement Science, Faculty of Medicine and Health Sciences, Norwegian University of Science and Technology, 7491 Trondheim, Norway

**Keywords:** Power, Kinematics, Metabolic cost, Fenn effect

## Abstract

In many whole-body exercise forms, e.g. cycling, the relationship between metabolic rate (MR) and mechanical power output during exercise is linear and unique. Such linearity is not seen for walking. We investigated whether total muscle power (*P*_tot_), i.e. power required for the inverted pendulum motion in walking plus power against net external resistance, demonstrated such a single relationship between *P*_tot_ and MR, independent of walking conditions. We studied walking under conditions in which considerable net external work against the environment was done (walking uphill and when resistive forces are imposed) as well as on the level, i.e. without net external work done. Fifteen adults walked freely on a large treadmill at 27 combinations of three speeds (3, 4.5 and 6 kmh^−1^), three inclines (0%, 3% and 6%) and three resistive forces (0.1×9.81, 2.5×9.81 and 5×9.81 N). Kinematics were recorded by motion capturing. MR was estimated from gas exchange recordings. Required *P*_tot_ generated by skeletal muscle was estimated as the power associated with step-to-step transitions (*P*_sst_) in addition to net external power (*P*_ext_), with *P*_tot_=*P*_ext_+*P*_sst_. The *P*_ext_*–*MR relationship was not entirely unique (*R*^2^=0.883) and was strongly affected by speed (*P*=0.004). The *P*_tot_*–*MR relationship was stronger (*R*^2^=0.972) and the influence of walking speed was almost cancelled out. The *P*_tot_*–*MR relationship resembles that found for cycling. On that basis, we conclude that *P*_tot_ seems to incorporate the major amount of work done during walking.

## Teaser

**Summary:** A model explaining work done by skeletal muscle in walking indicates that work against environmental forces and work required for the inverted pendulum motion determine required work during walking.

## INTRODUCTION

The metabolic cost of walking has been a topic of scientific interest for decades. As walking is the major mode of locomotion for humans, a fundamental understanding of what makes walking cost metabolic energy is obviously of importance, particularly for applied fields dealing with cases in which walking becomes strenuous (e.g. rehabilitation, geriatrics).

A linear relationship between total cost and external work done (i.e. work on the entire body as represented by the centre of mass, CoM) against external forces is observed, for example, in cycling (e.g. Ettema and Lorås, 2009; McDaniel et al., 2002), cross-country skiing (e.g. Hegge et al., 2016; Sandbakk et al., 2010), swimming (Toussaint et al., 1990) and wheelchair propulsion (Puchinger et al., 2022). This is not surprising because, besides the cost of generating force (Griffin et al., 2003), producing work is a predominant determinator for the metabolic cost of muscle contraction, both *in vitro*, i.e. the Fenn effect (Fenn, 1923, 1924; Mommaerts, 1970; Rall, 1982) and *in vivo* (Ortega et al., 2015). Fenn (1923, 1924) demonstrated that, for isolated muscle, the total cost of concentric muscle contraction is described by a linear relationship between cost and work done (*E=I+kW*, Fenn effect), where *E* is the total cost, *I* is the cost of isometric force generation, *W* is the work done and *k* is the slope of the linear relationship. We suggest that if one can identify all work done by muscle during whole-body movement, a similar linear relationship (Fenn effect) between metabolic cost and total work emerges.

Of course, in whole-body exercises, other processes than doing work and generating force (e.g. breathing and circulation) may affect the metabolic cost regardless of whether they are constant or their intensity is directly linked to doing work (see Ettema and Lorås, 2009). However, while the cost of such processes may depend on work rate, they are unlikely to depend on loading conditions. When walking freely with a self-chosen technique, this independence would also apply to factors that affect the metabolic cost of doing work, e.g. muscle fascicle length (Beck et al., 2022), muscle shortening velocity (Swinnen et al., 2023) and activation dynamics (Hogan et al., 1998).

Thus, while the Fenn effect emerges – at least phenomenologically – in many whole-body exercises, in walking this is not apparent. Even when level walking without any form of external resistance and thus not doing any net external work on the CoM (over an entire stride), the energy cost is far above the resting cost and strongly depends on walking speed (Adeyeri et al., 2022; Ralston, 1958). This is unlike other locomotion forms. For example, in cycling, pedalling at typical cadences without any external resistance is done at hardly any extra metabolic cost (e.g. Foss and Hallén, 2004; Tokui and Hirakoba, 2007, 2008). Evidently, in walking, irrespective of external resistance, the locomotor mechanism itself demands muscles to spend considerable energy and do work despite the fact that no net external work is done. Beside body support (Grabowski et al., 2005) and the motion of body segments (particularly the lower limbs) (Gottschall and Kram, 2005), the cost of step-to-step transitions (Donelan et al., 2002a,b; Kuo et al., 2005) is likely to be an important contributor to this cost in walking. The latter contributor is velocity dependent and is a direct result of the classic inverted-pendulum mechanism that applies to walking (Cavagna et al., 1963, 1976). In the hanging pendulum, the dynamics are smooth, i.e. the velocity (and associated kinetic energy) smoothly reduces to zero – and is inverted afterward – while height (and associated potential energy) increases to a maximum value reciprocally. In the inverted pendulum, this turn-around happens at heel strike collision and is abrupt. Most importantly, the velocity changes direction instead of being abolished and consequently reversed (Donelan et al., 2002a,b; Kuo et al., 2005). This redirection of velocity entails an impulse-induced velocity reduction (without a reciprocal change of height) and thus loss of kinetic energy at each step that is not recovered by the increase of potential energy. This amount of energy, i.e. work for the step-to-step transition (*W*_sst_), must be delivered by muscle of the contra-lateral limb (Donelan et al., 2002a,b; Kuo, 2007; Kuo et al., 2005). The *W*_sst_ estimate is based on a direct work (power) identification, i.e. on a displacement (velocity) and a force vector (Donelan et al., 2002a; Kuo et al., 2005), and verified empirically using full dynamics recordings (Donelan et al., 2002b). There is no indication that, in walking, the energy lost in this transition is stored in an elastic or other form to be re-utilised later (Donelan et al., 2002a). The amount of *W*_sst_ can be deduced from the global kinematics of walking. The power form of *W*_sst_ (i.e. *P*_sst_) depends on lower limb length, step length, CoM velocity and step rate.

In many other locomotion modes, e.g. cycling, cross-country skiing, skating, such constraints of landing impact and associated mechanisms that reduce the body's mechanical energy are not apparent. Therefore, it may well be that the work required to handle step-to-step transitions explains why a unique power–metabolic rate (*P*–MR) relationship seemingly does not exist for walking, while it does for these other locomotion modes.

The step-to-step transition work concept has been validated for metabolic cost prediction regarding, for example, the effects of step length (and frequency) and width (Donelan et al., 2001; Kuo et al., 2005), over a range of speeds (Donelan et al., 2002b), and for amputee walking (Houdijk et al., 2009). Yet, it has rarely been tested in a net external *P*–MR perspective, i.e. in combination with doing considerable net external work on the CoM. Neither are we aware of studies that explicitly examine the walking speed effect in this regard. Net external work (*W*_ext_), i.e. work done on the CoM by external resistance forces, during level walking over an entire stride is negligible (not to be confused with positive work done during push-off). However, considerable net external work is done in, for example, uphill walking or when any form of external resistance is imposed constantly. Even though the metabolic cost of walking on inclines has been studied extensively, little is known about whether the cost for external work depends on the manner in which external work requirements are imposed (see Parker et al., 2005). For example, imposing resistive force in level walking may lead to slightly different work–cost relationships from those for uphill walking (see Parker et al., 2005). Furthermore, to our knowledge, no attempt has been made to assess whether a single linear relationship between total work done and metabolic cost can be established for walking, with and without external resistance.

In the case that indeed all muscle work done is reflected in the required *W*_sst_ (linked to the walking mechanism) and the external work done, this should lead to a single work–metabolic cost relationship (i.e. Fenn effect), independent of the walking condition (including walking speed). We tested this idea by assessing metabolic cost and estimating required work during walking at different speeds, inclines and external resistance (via a pulley system). In this study, the setup was restricted to freely chosen, preferred walking step length and frequency. We compared two models: one using *W*_ext_ as baseline and a second adding *W*_sst_ to *W*_ext_, and compared their relationship with metabolic cost. By applying an integrated approach (i.e. using measures for entire strides and total metabolic cost), we avoided the interpretation challenges when using positive and negative work during each push-off and landing, respectively, to estimate total work done by muscle (e.g. Donelan et al., 2002b; Ettema, 2001; Prilutsky, 1997; Umberger and Martin, 2007).

## MATERIALS AND METHODS

### Participants

Fifteen adults (mean±s.d. 32±8.3 years, 78.1±15.8 kg, 1.79± 0.09 m, 11 men, 4 women) participated in the study. This study was approved by the Norwegian Centre for Research Data (ref. no. 761888). Prior to testing, informed written consent was obtained from each participant. They were informed about the aim of the study in general terms, and that they could withdraw unconditionally at any point.

### Experimental protocol

All experiments were conducted at the NeXtMove core laboratory facility, Norwegian University of Science and Technology (NTNU). All tests were performed on a large treadmill prioritising the combined measurements of gas exchange (for MR) and kinematics (for mechanical power), under an unrestricted walking technique. The lack of force plates made a detailed dynamics analysis impossible. The participants were asked to walk freely on a large motorised (5×3 m) treadmill (Forcelink Technology, Zwolle, The Netherlands) under different conditions. This included a brief familiarisation period. That is, the participants walked for a few minutes under a selected number of conditions, including pulley weight, incline and high speed, but never for a shorter duration than they indicated to be comfortable. Furthermore, they experienced the use of a mouth-piece and nose-clip for gas exchange measurements while walking before formal testing. The conditions consisted of all combinations of three speeds (3, 4.5 and 6 km h^−1^), three inclines (0%, 3% and 6%) and three resistance weights (0.1, 2.5 and 5 kg) on a rope guided through a pulley system (Ettema et al., 2023). In the unloaded condition, 0.1 kg instead of 0 kg was used to avoid slack in the rope. This resulted in a small external power (0.8, 1.2 and 1.6 W at 3, 4.5 and 6 km h^−1^, respectively) for this condition at zero incline. For all combinations, the participants indicated that they were walking naturally rather than experiencing a forced deviating locomotor technique. Each combination resulted in a predetermined external power to be delivered by the participant (see Eqn 2, below). Thus, total walking under 27 external conditions was executed.

**Fig. 1.**
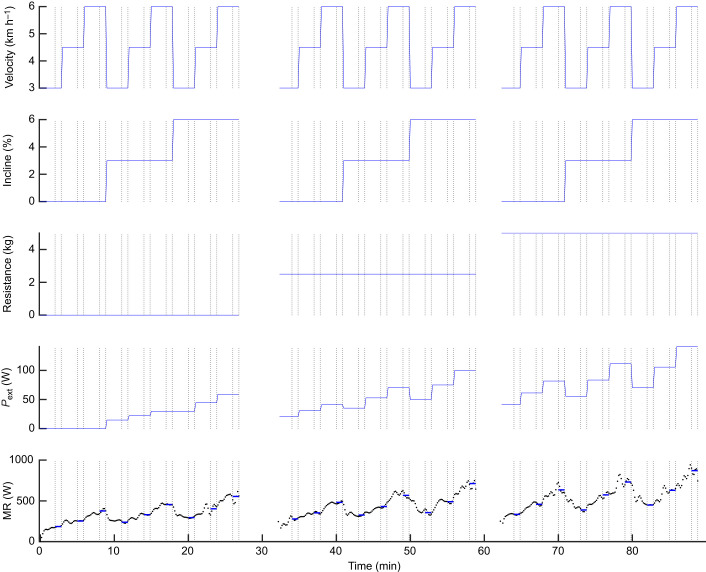
**Experimental protocol.** The protocol consisted of 27 combinations of walking velocity (3, 4.5 and 6 km h^−1^), incline (0%, 3% and 6%) and resistance (0.1, 2.5 and 5 kg). An example of external power (*P*_ext_) and metabolic rate (MR) for one participant is also shown. Periods without traces are rest periods between the three resistance blocks. Vertical dotted lines indicate steady intervals from which data were retrieved for further analysis. Horizontal blue lines are the mean values of MR for these periods.

For practical reasons as well as to ensure that the conditions with highest power were done toward the end of the protocol – thus minimising potential fatigue effects – the order of conditions was not randomised (Fig. 1). One protocol session consisted of 9 speed–incline ordered combinations with one specific pulley weight: lowest speed with three incremental inclines (0%, 3%, 6%), middle speed with same ordered inclines, followed by highest speed for the three same inclines. This protocol session was then repeated with the next pulley weight. Each speed–incline–weight condition lasted 3 min, directly followed by the next condition of velocity or incline. Thus, power increments were small, and steady state (i.e. such that, apart from inherent variation because of the breath-to-breath characteristic of respiration, respiratory variables showed no obvious trends) was typically obtained within about 2 min at each stage (Fig. 1), as also shown by Adeyeri et al. (2022). When changing the pulley weight, a minimum of ∼2 min rest period was incorporated.

### Equipment and measurements

Total MR was calculated according to Peronnet and Massicotte (1991), based on gas exchange variables (*V̇*_O_2__ and respiratory quotient – mean of the last minute of each condition), which were acquired through open circuit indirect calorimetry (Vyntus CPX, Vyaire Medical, Mettawa, IL USA).

Kinematic data were obtained at 100 Hz sample rate using 3D motion capture (Oqus, Qualisys AB, Gothenburg, Sweden). Reflective markers were placed on 15 bony landmarks (base of 5th metatarsal, lateral malleolus, lateral epicondyle of femur, greater trochanter, 7th cervical vertebra, lateral edge of acromion process, lateral epicondyle of humerus and styloid process of radius; except for cervical vertebra, all bilateral) to estimate the location of the body's segments. The estimation of a segment’s mass, its location and moment of inertia were done according to de Leva (1996), from which the CoM position and its velocity (*v*_CoM_) were estimated (e.g. Danielsen et al., 2019). Markers of the lower extremity were used to estimate variables required for the estimation of *P*_sst_ according to Kuo et al., 2005 (their equation 3) and accounting for step frequency:
(1)

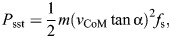
where α is half of the angle between the trailing (hip–toe) and leading (hip–ankle) limbs at touch-down and *f*_s_ is step frequency. Note that using this approach, the issue regarding differences between the ‘individual limb’ or ‘combined limb’ method for *P*_sst_ estimation (Donelan et al., 2002b) is not relevant. Net external power (*P*_ext_) was found similar to our previous method (Ettema et al., 2023):
(2)

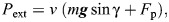
where *v* is the walking velocity, γ is the incline, *m* is body mass, ***g*** is gravitational acceleration and *F*_p_ is the resistance force by the pulley system (the estimation of *F*_p_ on the basis of pulley weight in this setup was validated previously; Ettema et al., 2023). Both equations estimate net power over the entire (series of) strides, and their sum:
(3)

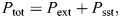
is the minimum total power (*P*_tot_) that must be delivered by muscle. It does not include any other potential additional work done in a stride during push-off (see Discussion).

### Statistical analyses

Linear regression was applied to the *P–*MR relationships. Identification of the effect of condition factors (velocity, incline and pulley resistance) was done according to Crowder and Hand (1990): the residual sum of squares for regression of all data (pooled – 27 data points per participant) was compared with the total residual sum of squares of three separate regressions on data stratified according to one condition factor (velocity, incline and resistance; nine data points for each of the three separate regressions). A general linear mixed model was not applied because of the entangled but direct effects of these condition factors on power (Eqns 1–3) and thus MR. Furthermore, the central issue was to investigate which power (Eqns 2 and 3) would lead to the best fit of the data on a single straight line (smallest residual sum of squares for pooled regression), i.e. potentially representing total muscle power and least affected by any condition factor.

An ANOVA for repeated measures was used to describe the dependence of *P*_sst_ on protocol factors (velocity, incline and resistance).

## RESULTS

**Fig. 2.**
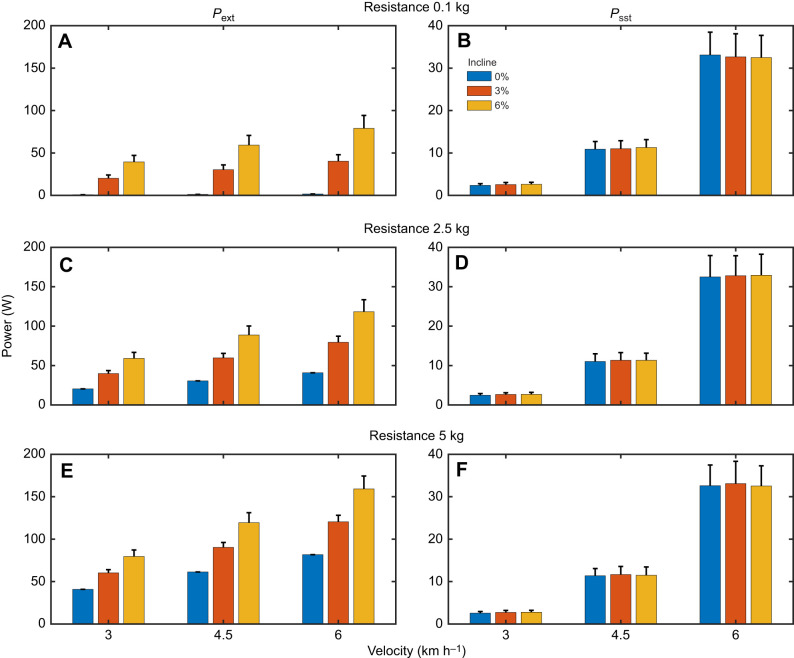
**Power components for the 27 condition combinations organised according to resistance, incline and velocity.** *P*_ext_ (left) and *P*_sst_ (power for the step-to-step transition; right) are shown for a resistance of 0.1 kg (A,B), 2.5 kg (C,D) or 5 kg (E,F) and incline of 0%, 3% and 6% (indicated by the colour signature in the key) (means±s.d., *n*=15). Note that *P*_ext_ was determined by protocol only.

The power contributions (*P*_ext_, *P*_sst_) are presented in Fig. 2. *P*_ext_ depended on all factors of the protocol and ranged from 1 to 160 W; *P*_sst_ depended only on velocity (*P*<0.001, η^2^=0.98) and ranged from 2 to 33 W. The associated kinematic variables, α and *f*_s_ (Eqn 1), depended mostly on velocity (*P*<0.001, η^2^=0.960 and 0.97, respectively), and far less on incline and resistance, albeit significantly (*P*<0.001, η^2^≤0.01 for all). In other words, all three condition factors affected the walking pattern, but only velocity did so to a substantial and meaningful extent. Note that *v*_CoM_ was not considered here because of its obvious dependence on the velocity condition factor.

**Fig. 3.**
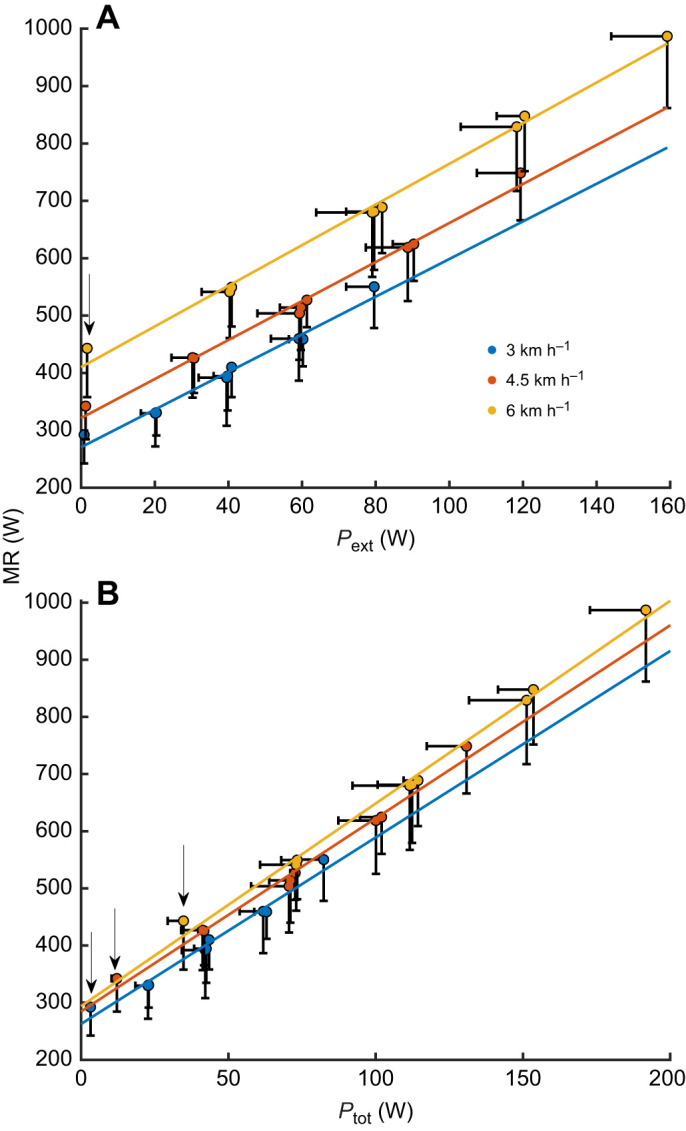
**Power–MR regressions of population mean data, stratified for velocity condition.** (A) *P*_ext_. (B) *P*_tot_ (total power). Data are means±s.d. (*n*=15). Vertical arrows indicate the conditions with *P*_ext_≈0.

Fig. 3 shows MR against *P*_ext_ and *P*_tot_ and stratified according to walking velocity (by colour signature) for the mean data (individual data are shown in Figs S1 and S2). Table 1 provides the statistical outcome of the regression and stratification (on all individual data). The pooled *P–*MR regressions show high *R*^2^ values, i.e. in all regression comparisons, power explains most of the MR variation (when doing external work at a rate of up to 160 W). Nonetheless, velocity has quite a strong impact on the *P*_ext_ *–*MR relationship (10% of MR variation explained by it, *P*=0.004). In contrast, the effect of velocity strongly diminishes in the *P*_tot_ *–*MR relationship (i.e. *P*_tot_ explains 97.2% of the MR variation, velocity explains an additional 1.2%, *P*=0.082). Note that the three conditions with *P*_ext_≈0 (indicated by a vertical arrow in Fig. 3) are aligned close to the general trend of the data for *P*_tot_, but not for *P*_ext_. The impact of incline (and resistance) is much weaker (statistically non-existent). These findings show that enhanced MR in walking is also present while doing external work, is velocity dependent, and can be explained by muscle work required for step-to-step transitions.

**Table 1.** *R*^2^ of the power–metabolic rate linear regression, pooled and accounting for condition factors

Power	*R* ^2^				*P*		
	Pooled	Stratified condition			Stratification effect		
		Velocity	Incline	Resistance	Velocity	Incline	Resistance
*P*_ext_	0.883	0.983	0.908	0.906	0.004	0.291	0.378
*P*_tot_	0.972	0.984	0.980	0.979	0.082	0.174	0.262

*P*_ext_, external power; *P*_tot_, total power.

All individual *P*_ext_ *–*MR and *P*_tot_*–*MR data are shown in Figs S1 and S2. These indicate that all subjects show similar MR responses, particularly regarding the velocity impact.

## DISCUSSION

We examined whether a model for the energetic cost of step-to-step transitions in walking applies to walking conditions during which extensive work is done against external resistance. In our experiments, a MR was achieved of up to 900 W in comparison with 300–450 W in unloaded walking. One main finding is that walking velocity and doing considerable external work influence the cost of walking independently of each other. The velocity effect on the *P–*MR relationship seems reduced to a great extent when accounting for step-to-step transition work. By estimating mechanical muscle work done in walking by adding the required costs due to step-to-step transitions (Donelan et al., 2002a,b; Kuo et al., 2005), a single linear *P–*MR relationship, almost independent of velocity and explaining more than 97% of the variance in MR, was found. The findings confirm results for unloaded walking, i.e. at zero net external work (Donelan et al., 2002b), and that the cost of the inverted pendulum mechanism in walking is independent of the net external work done. The current study was restricted to freely chosen step frequency and length, probably minimising the step-to-step transition cost. We warn against generalisation to non-optimal walking conditions. Any indication of the effect of such conditions requires additional studies.

### Velocity versus incline and pulley resistance

We treated the loading condition factors (velocity, incline, pulley resistance) the same in the statistical analysis, but the physical meaning of the factors is clearly different. While the effect of velocity on metabolic cost is – partly – independent of whether or not external work is affected (e.g. Adeyeri et al., 2022; Cavagna and Kaneko, 1977; Mian et al., 2006; Piche et al., 2022; Ralston, 1958), the effects of incline and resistance are mostly – if not entirely – because they influence external work to be done, at least within the range of inclines used here (e.g. Minetti et al., 1993). The current results on the movement kinematics support this notion, as explained here. The additional power (*P*_sst_) is based on the kinematic data. Apart from *v*_CoM_, which logically is closely related to the walking speed, *P*_sst_ depends on stride length (mostly determined by α) and *f*_s_ (Eqn 1). Both, when walking freely, depend strongly on walking speed, and only to a very small extent on incline and resistance. In other words, the walking pattern and related *P*_sst_ depend mostly on walking speed, not on doing net external work.

### Does step-to-step transition work entail all muscle work beyond external work?

**Fig. 4.**
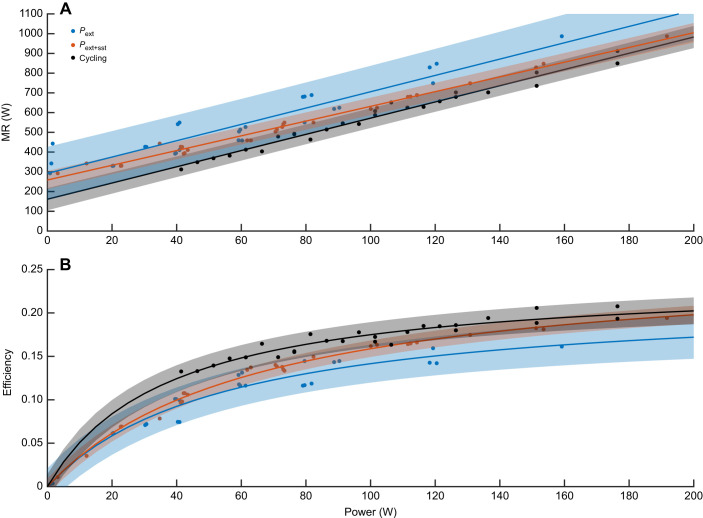
**MR and efficiency against two estimates of total power, compared with cycling.** (A) MR. (B) Efficiency (*P*/MR). Total power was estimated as *P*_ext_ and *P*_ext_+*P*_sst_. Cycling data are from Bardal et al. (2013) and unpublished data from our laboratory (G.E. and K.S.). Shaded areas indicate 95% confidence bands (*n*=15).

We argue that in free walking, people tend to choose a mode (e.g. step size and frequency) that minimises metabolic cost and that most of the total metabolic cost, beyond resting baseline, depends on mechanical work to be done by muscle. Thus, a model that correctly estimates the total amount of muscle work should lead to a *P–*MR relationship independent of any external condition factor (velocity, incline, resistance). The current results indicate that the step-to-step transition concept indeed leads to such an outcome, but this is not ‘full proof’ that the model accounts for all – or at least most – work done by muscle. As proof of concept, we therefore compared our findings with similar data for ergometer cycling, obtained from Bardal et al. (2013) and our own lab (G.E. and K.S., unpublished). In seated (ergometer) cycling, little extra metabolic cost is required for static muscle action, e.g. for maintaining posture, and very little muscle work is done beyond external work as recorded at the ergometer (Foss and Hallén, 2004; Minetti, 2011; Minetti et al., 2020; Tokui and Hirakoba, 2007, 2008). External power in ergometer cycling, adjusted for ∼2 W for internal losses, can be regarded as a decent estimate for total muscle mechanical power (see Minetti, 2011; Minetti et al., 2020). The current *P*_tot_ results for walking show – at least by approximation – a similar *P–*MR relationship to that for cycling (Fig. 4A). Fig. 4B also shows corresponding efficiency (*P/*MR)–*P* relationships.

Both walking *P–*MR relationships show a higher cost and lower efficiency than cycling. However, the *P*_tot_ relationship approaches that for cycling at higher power range (>∼140 W). In other words, this comparison indicates that the cost of the step-to-step transition, besides external work, accounts for much of the extra work (to be) done by muscle. Still, especially at low mechanical power, for a given amount of muscle power, walking comes at a higher cost. One mechanism that requires metabolic cost but does not necessarily involve muscle work is maintaining upright posture (compared with sitting in cycling), but this may explain only about 5 W of the metabolic cost difference (Júdice et al., 2016). Moreover, the step-to-step transition model does not include cost of the swing motion of the lower extremities. Based on findings by Gottschall and Kram (2005) and Doke and co-workers (Doke et al., 2005; Doke and Kuo, 2007), this would amount to an additional MR of about 50–60 W in our study. Together, these costs explain a major part of the walking–cycling MR differences.

The improvement by including *P*_sst_ in muscle power estimation (Eqn 3 versus Eqn 2) of MR prediction may not seem surprising given the strong dependence of *P*_sst_ on velocity by definition (Eqn 1) and MR dependence on walking velocity (Adeyeri et al., 2022; Ralston, 1958). However, the key issue is not just a good correlation (in the current setup, *P*_ext_ already explains a major amount of the variation in MR) but that the data obtained under different loading conditions match a single linear curve (Fenn effect). Thus, even though the *R*^2^ values (Table 1) are of course strongly indicative of this, the improved alignment of three velocity data lines as shown in Fig. 3B versus 3A is essential for this interpretation (and is indicated by *P*-values of velocity stratification). It must be noted that we only analysed the original and simplest version of the step-to-step transition model. Any deviation of the model (Eqn 1) from actual kinetics (Adamczyk and Kuo, 2009) is not accounted for. For example, the assumed instantaneous change of velocity is not correct (Adamczyk and Kuo, 2009). Furthermore, the model does not include any effect of tendon elasticity (see below). Thus, while the model (Eqn 1) seems quite robust in form, its parameter values are debatable (Adamczyk and Kuo, 2009; Kuo et al., 2005). To investigate whether changing parameters in the model would improve the alignment, we re-analysed the data adding a constant *K* to Eqn 1:
(4)

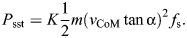
A value of *K*=1.5 improved the model most with *R*^2^=0.979 (*P* of velocity stratification=0.37). A larger value of *K* worsened the prediction, i.e. the data for the three velocities diverged from each other in opposite directions (as compared with Fig. 2A). That is, the current prediction model ‘lacks’ maximally about 15 W (*K*=1.5: 30 W raised to 45 W) at the highest walking speeds, 5 W at intermediate speed and 1–2 W at the lowest speed. In other words, the original and simplest model does not entail all velocity-related required muscle power. Some explanations are briefly discussed in the next sections.

In this study, we did not include the participants' *P–*MR outcome for cycling and made no direct statistical paired comparison. We instead opted for comparison with existing data because cycling *P–*MR relationships are very robust (Ettema and Lorås, 2009). The two cycling datasets used here (Bardal et al., 2013; and G.E. and K.S., unpublished) show close similarity. Furthermore, it was not our aim to identify the exact differences between the walking and cycling curves but rather to use cycling as a reference benchmark for which, as explained above, it is reasonable to suggest that (almost) all work done by muscle is captured as work measured at the crank (or any other site on the bicycle/ergometer). We emphasise that we consider the similarities between the *P–*MR relationship for walking and cycling to be nothing more than proof of concept, showing that the muscle power estimates based on the step-to-step transition work concept are realistic. In this regard, it is maybe even just as important to note that the model predicts walking efficiency that does not exceed cycling efficiency (which would indicate overestimating muscle work).

As mentioned, the findings in this study are a proof of concept for the step-to-step transition model under conditions in which considerable external work is done, but, so far, only using preferred walking technique. It does not disqualify any other model for estimation of muscle work. For example, the internal work approach (e.g. Cavagna et al., 1976; Peyré-Tartaruga et al., 2021) estimates additional (‘internal’) muscle work based on kinetic energy exchange of body segments. Even though it has been criticised on fundamental grounds (Aleshinsky, 1986; van der Kruk et al., 2018; van Ingen Schenau, 1998; Zatsiorsky, 1998), it may predict just as well as the step-to-step transition concept. A main challenge for the internal work approach may be that total work is overestimated, leading to unrealistic efficiency (Ettema, 2023). Models that focus on the cost of force production rather than doing work (e.g. Grabowski et al., 2005; Griffin et al., 2003) may be equally successful. In our opinion, success would not be surprising: apart from pure isometric contraction for maintaining posture and control, in walking, power and force production are fully intertwined. Thus, detailed comparisons of such approaches may be fruitful to identify overlap and essential differences between the theories behind the models.

### Role of elastic energy

The possible role of storage and release of elastic energy is a mechanism that is usually considered when explaining metabolic cost in walking and running. This mechanism is not incorporated into the step-to-step transition concept, and we did not take this mechanism into consideration in the data analysis because, in fact, it is irrelevant for the purpose of this study, i.e. validating a model for estimating total muscle work: if indeed all work done by muscle is accounted for, it does not matter where this work ‘ends up’. It may well be first stored as elastic energy before being used in propulsion, and thus turned into external work with delay. Such delay does not affect the *P–*MR relationship. For a particular exercise, this mechanism of storage and release of elastic energy very likely keeps muscle work and its metabolic cost at a reduced level, thereby minimising the reduction of gross efficiency at the organismal level (i.e. *P*_ext_*/*MR not *P*_tot_*/*MR) below muscular efficiency.

In the case that a part of *W*_sst_ is stored elastically, reutilised and consequently emerges as *W*_ext_, the model overestimates total muscle work. Even though some empirical evidence indicates otherwise (Donelan et al., 2002a), this possibility should be kept in mind. In such a case, i.e. the current model overestimating total work done by muscle, the unexplained discrepancy between walking and cycling increases.

As a sidenote, irrespective of any beneficial function, the storage and release of energy is a necessity because muscles must build up force and relax afterward during each stride. This process implicates storage and release of elastic energy. As mentioned, it may also merely imply delaying, possibly smoothing – but not enhancing – the total propulsion action by muscle over an entire stride (Ettema, 2001; Griffiths, 1989). Thus, in walking, the benefits of this process may be other than conserving energy; for example, optimising fascicle state, i.e. length and velocity (Ettema, 2001; Lichtwark and Barclay, 2010). One may even speculate whether there are any benefits at all: while consensus exists about the beneficial role of series elastic structures in running, in walking, we may simply manage the consequences of their existence.

### Other factors of relevance

Factors that affect metabolic cost, such as muscle length, shortening velocity, activation dynamics and force generation, may have differed depending on the conditions in the present study. Thus, these may have affected the *P–*MR relationship. Still, MR was almost entirely explained by *P*_tot_. This indicates that such factors were similar over conditions or relate to a relatively constant cost (affecting the intercept), or the cost changes according to power (affecting the slope). Beck et al. (2022) and Swinnen et al. (2023) demonstrated clear effects of respectively muscle length and shortening velocity on metabolic cost. However, their studies were designed to elucidate the mechanisms and therefore comprised length and velocity differences that are rather large compared with what is feasible in free walking. Furthermore, the length excursions and velocities were all below optimum values, which magnifies effects compared with those around optimum settings. Thus, these effects were probably much smaller in our study. Still, should length and velocity differences have played some role of importance, they would have been included in *P*_sst_ because step length and step frequency directly affect all these variables. Thus, these factors may influence the slope of the *P–*MR relationship.

Isometric force generation (e.g. for upright posture) is often considered as a cost factor as, for example, by Hoogkamer et al. (2014) in running. We conducted some additional experiments on four participants adding 10 kg in a backpack at 4.5 km h^−1^ at all three inclines and 0 and 5 kg pulley weight conditions. Inclusion of these data did not change the major outcome. Still, for level walking without pulley resistance, any additional metabolic cost beyond what is predicted by the step-to-step transition cost could be attributed to extra force required to hold the extra mass. The outcome of these additional tests suggests that indeed about 50 W extra metabolic cost may be related to isometric force generation.

## Supplementary Material

10.1242/jexbio.250352_sup1 Supplementary information
