# Supplementary material for: On the estimation of total muscle work done in human walking
Source: J Exp Biol. 2025 Jun 13;228(12):jeb250352. doi: 10.1242/jeb.250352 (PMC12211589; doi:10.1242/jeb.250352)
Supplement: Supplementary information [file jexbio-228-250352-s1.pdf]

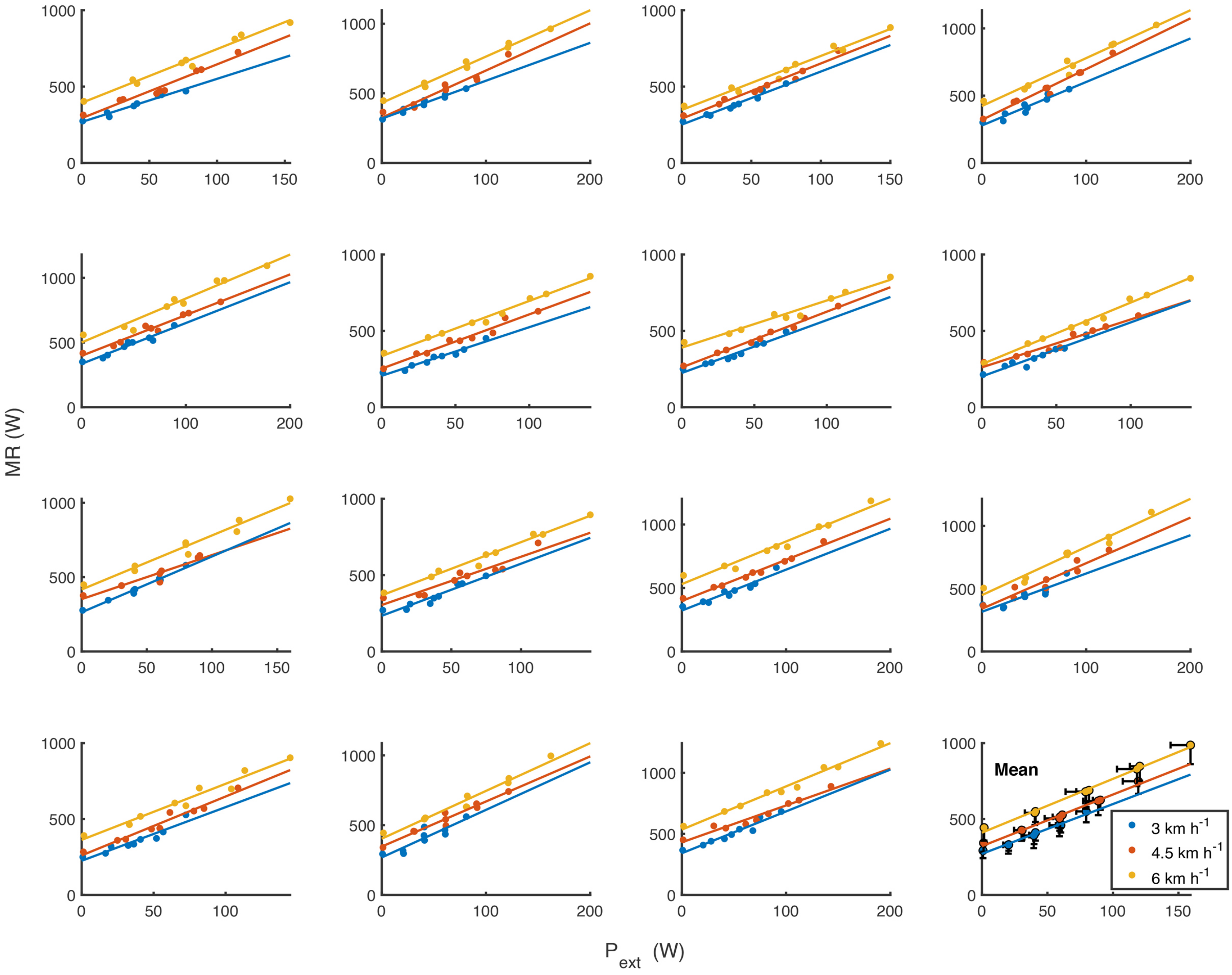


**Fig. S1.** Regression of individual $P_{ext}$ – *MR* data. Last subplot (mean) is a replication of Fig. 3A for reference.

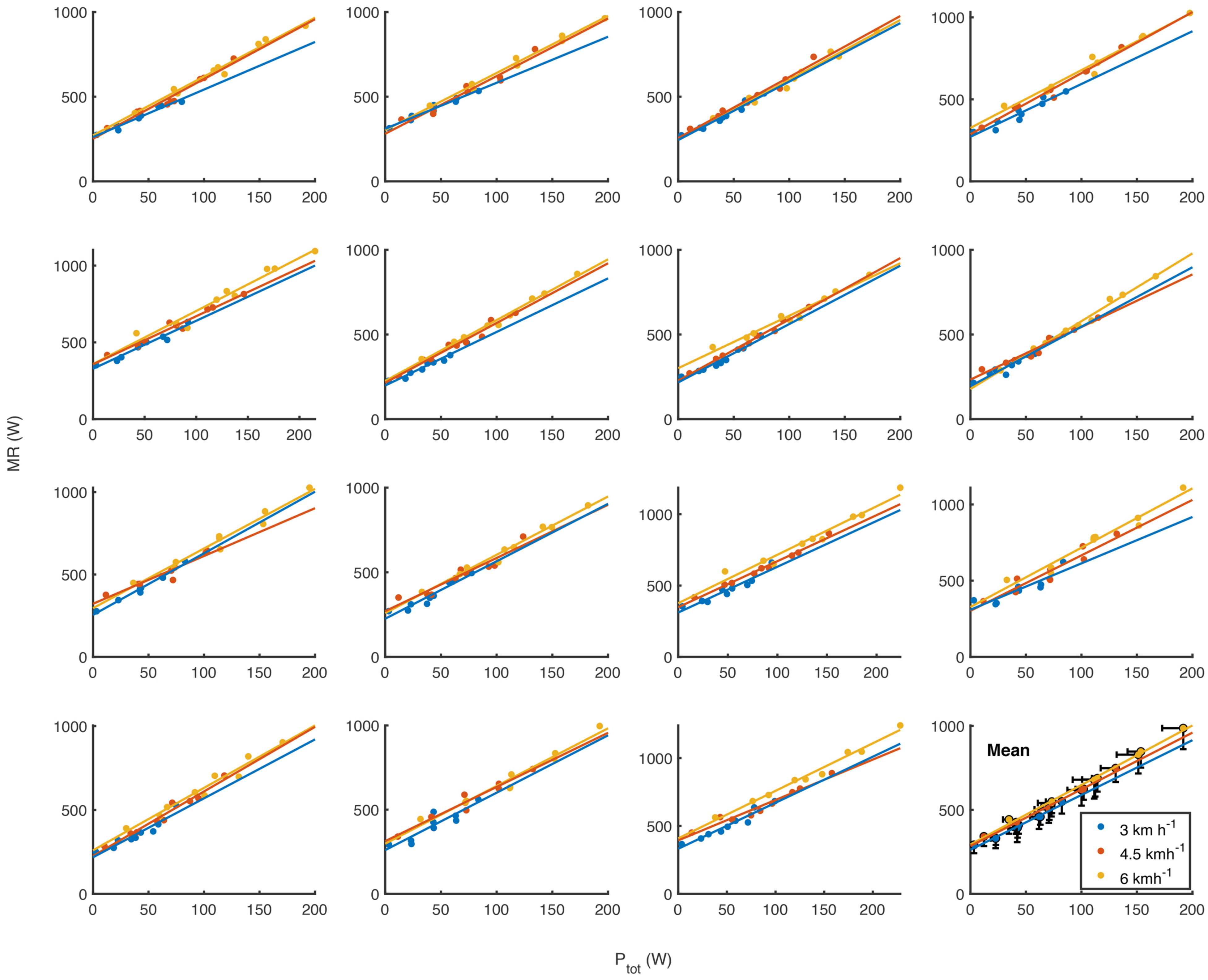


**Fig. S2.** Regression of individual $P_{tot} - MR$ data. Last subplot (mean) is a replication of Fig. 3B for reference.

Journal of Experimental Biology • Supplementary information
